# Electrocortical correlations between pairs of isolated people: A reanalysis

**DOI:** 10.12688/f1000research.11537.1

**Published:** 2017-05-15

**Authors:** Dean Radin

**Affiliations:** 1 Integral and Transpersonal Psychology, School of Consciousness and Transformation, California Institute of Integral Studies, San Francisco, CA, 94103, USA

**Keywords:** electrocortical, coherence, synchronization

## Abstract

A previously reported experiment collected electrocortical data recorded simultaneously in pairs of people separated by distance. Reanalysis of those data confirmed the presence of a time-synchronous, statistically significant correlation in brain electrical activity of these distant “sender-receiver” pairs. Given the sensory shielding employed in the original experiment to avoid mundane explanations for such a correlation, this outcome is suggestive of an anomalous intersubjective connection.

## Introduction

Giroldini *et al.* (2016) reported an experiment where pairs of people isolated by distance each had 14-channel electroencephalograms (EEGs) recorded simultaneously (Emotiv EPOC+, San Francisco, CA). The “sender” (S) of each pair was exposed to 128 stimulus epochs per test session, where each epoch consisted of a one-second exposure to a light or sound stimulus (the latter presented over earbuds). Using a whole brain EEG coherence metric, Giroldini *et al*. found that after 25 experimental sessions that the “receiver’s” (R) electrocortical coherence increased during the stimulus epochs. This was interpreted as a reflection of a “nonlocal” connection between S and R. The effect was primarily observed in the EEG alpha band of 8 – 12 Hz, with a statistically stronger effect reported in the range of 9 – 10 Hz. To double-check how robust the reported effect might be, this study developed a simpler correlational approach and applied it to the original, unfiltered EEG data.

## Methods

The raw EEG data from Giroldini *et al.* (2016) was obtained from: doi, 10.6084/m9.figshare.1466876.v8 ( Tressoldi, 2016).

Matlab (R2013a) scripts were written to conduct the analysis. These scripts may be obtained from: 10.6084/m9.figshare.4954643.v2 ( Radin, 2017).

To process the raw EEG data, first use the script readEEG.m (this uses the function importfile1.m), then put all of the newly processed files (in Matlab’s .mat format) into a single folder and run the script EEG_xcorr_raw.m in that folder. This will create Giroldini’s *et al*.’s brain coherence metrics for each pair of participants. Finally, run the script EEG_analysis_Radin.m, which will analyze those files and generate results in graph form.

As a brief description of the method, the processing scripts follow Giroldini *et al*.’s method for creating a whole brain coherence metric for each S and R datafile. The scripts then create an ensemble median of this metric plus and minus one second from stimulus onset. A Pearson correlation is then formed between the ensemble median curves for S and R pairs. The two-tailed p-value associated with that correlation is transformed into a one-tailed z score using an inverse normal transform. Then a nonparametric permutation analysis is used to determine the probability associated with that z score (i.e., this z is not distributed as a standard normal deviate because its variance is inflated due to the autocorrelated nature of EEG data). The p-value resulting from the permutation analysis is converted into a standard normal deviate (this is now a conventional z score). The same process is used on the remaining 24 pairs of EEG data. The final step combines the 25 z scores into a Stouffer Z = ∑z _s_/5, where Z is distributed as a standard normal deviate.

## Results

The above procedure results in a Stouffer Z = 2.705, p = 0.006 (two-tailed). Four of the 25 sessions are independently significant at p < .05 (two-tailed); all four of those sessions had positive S-R correlations.

**Figure 1.**
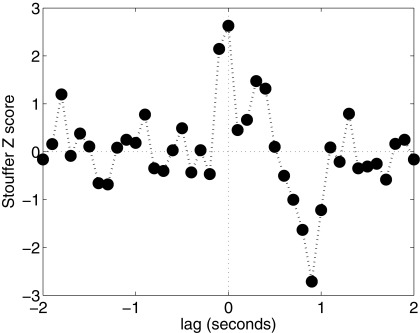
Time-synchrony analysis. Positive lags in this graph represent post-stimulus S-R correlations; negative lags are pre-stimulus.

To check if this S-R relationship is in time-synchrony, the Matlab script circular shifts each R’s EEG coherence signal -2 seconds, and then repeats the entire analytical procedure to determine the overall Stouffer Z score. Then R’s coherence signal is shifted to the right by 100 msec, reanalyzed, and this is repeated until reaching a lag of +2 seconds. If the original S-R correlation was synchronized in time, then we would expect to see the peak result at lag 0. Figure 1 shows that this was indeed the case. 

Figure 1 also shows a significantly negative deviation at a lag of 900 msec post-stimulus. Because this analysis is based on the absolute magnitude and not the direction of the correlation, this decline indicates that the S-R correlation strength declined *below* chance-expected levels about 1second post-stimulus. This may reflect a drop in electrocortical coherence in S generated by the explicit presentation of a stimulus; thus, during that time, the magnitude of the S-R correlation would be expected to momentarily drop. If similar negative correlations are observed in future experiments of this type, it may prove to be a useful secondary indicator of a genuine S-R relationship.

## Conclusion

Analysis of previously collected EEG data showed a significant time-synchronized correlation between the electrocortical activity of “sender” and “receiver” pairs. Because the data were collected under conditions where participants were isolated by shielding and distance, this outcome is suggestive of a “nonlocal” mind-to-mind interaction.

## Data availability

The data referenced by this article are under copyright with the following copyright statement: Copyright: © 2017 Radin D

Data associated with the article are available under the terms of the Creative Commons Zero "No rights reserved" data waiver (CC0 1.0 Public domain dedication). 

The raw EEG data from Giroldini *et al.* (2016) was obtained from: doi, 10.6084/m9.figshare.1466876.v8 ( Tressoldi, 2016).
