# Supplementary material for: Electrocortical correlations between pairs of isolated people: A reanalysis
Source: F1000Res. 2017 May 15;6:676. [Version 1] doi: 10.12688/f1000research.11537.1 (PMC5490474; doi:10.12688/f1000research.11537.1)
Supplement: Peer review history

# Peer review: Referee response for version 1

**DOI:** 10.5256/f1000research.12461.r22756

**Published:** 2017-06-23

**Authors:** Aliodor Manolea

**Affiliations:** 1 Faculty of Psychology and Social Sciences, Hyperion University from Bucharest, Bucharest, Romania

The statistical method seems to be the correct one if we consider each experimental session corresponding to an S-R pair as a separate experiment.

 The study is very concise and on the subject, and the results comes from  a logical thinking that is materialized in a mathematical method, perfectly adapted to the purpose pursued. Well done work.

I have read this submission. I believe that I have an appropriate level of expertise to confirm that it is of an acceptable scientific standard.

# Peer review: Referee response for version 1

**DOI:** 10.5256/f1000research.12461.r22753

**Published:** 2017-05-18

**Authors:** Edward Justin Modestino

**Affiliations:** 1 Department of Psychology, Curry College, Milton, MA, USA

This is a brief research note that is under review.  It refers to an independent reanalysis of data from another research group was done for a controversial study on non-local consciousness.  The reanalysis used a non-parametric permutation.  The only thing that I do not understand clearly is the results.  It appears that the results of 25 session (different subject pairs) divulged a significant p-value of p = 0.006 in a group analysis.  Next, it is explained that four out of the 25 sessions were independently significant at p<0.05 two tailed.  I am a bit confused.  I guess this means the greatest significance was seen at the group level, and at the subject level only four subject pairs showed significance.  I am not sure I am understanding this correctly.  Please make sure it is very explicitly stated to avoid the confusion I have had.

I have read this submission. I believe that I have an appropriate level of expertise to confirm that it is of an acceptable scientific standard.

# Response

**Published:** 2017-05-27

**Authors:** Dean Radin

**Affiliations:** California Institute of Integral Studies, USA

> It appears that the results of 25 session (different subject pairs) divulged a significant p-value of p = 0.006 in a group analysis.  Next, it is explained that four out of the 25 sessions were independently significant at p<0.05 two tailed.... I guess this means the greatest significance was seen at the group level, and at the subject level only four subject pairs showed significance.  

Yes, that is correct. The p value of p = 0.006 is a group analysis over all 25 sessions. When examining individual sessions 4 were independently significant at p < 0.05.  It is noteworthy that this latter outcome is unexpected by chance because the binomial probability of 4 or more significant (at p < 0.05) sessions out of 24 is associated with p = 0.03. What this suggests is that while some of the other sessions did not quite reach the (conventional) threshold for significance, on average they contributed results in the same direction, thus leading to the overall stronger statistical outcome for all data combined.
